# Current View on Genetic Relationships within the Bunyamwera Serological Group

**DOI:** 10.3390/v14061135

**Published:** 2022-05-25

**Authors:** Anna S. Dolgova, Marina V. Safonova, Oumar Faye, Vladimir G. Dedkov

**Affiliations:** 1 Saint Petersburg Pasteur Institute, Federal Service on Consumer Rights Protection and Human Well-Being Surveillance, 197101 Saint Petersburg, Russia; vgdedkov@yandex.ru; 2 Anti-Plague Center, Federal Service on Consumer Rights Protection and Human Well-Being Surveillance, 127490 Moscow, Russia; marina-iris@mail.ru; 3 Department of Virology, Institute Pasteur de Dakar, Dakar BP 220, Senegal; oumar.faye@pasteur.sn; 4 Martsinovsky Institute of Medical Parasitology, Tropical and Vector-Borne Diseases, Sechenov First Moscow State Medical University, 119435 Moscow, Russia

**Keywords:** Bunyamwera serogroup, *Orthobunyavirus* genus, ICTV

## Abstract

The Bunyamwera serological group includes a number of geographically widespread viruses that are related but not identical and have serological cross-reactivity. As the first group members were obtained in the pre-sequencing era, their classifications (group attribution, species differentiation) were originally based on serological reactions. At the same time, the accuracy of the typing in each case depended on the variety of viruses that the researcher had as a comparison panel. With the advent of sequencing techniques, it has become customary to use identity thresholds (nucleotide or amino acid composition) as demarcation criteria for the interspecific differentiation of viral species. Identity thresholds are determined by the International Committee on Taxonomy of Viruses (ICTV) and are regularly reviewed. Similar criteria were established for the *Orthobunyavirus* genus, which includes members of the Bunyamwera serological group. On the basis of these criteria, the species attributions of some members of the serological group need to be clarified. For this purpose, we analyzed sequences (available in NCBI GenBank) of viruses belonging to the Bunyamwera serological group in order to clarify their phylogenetic positions on the basis of the current demarcation criteria established by the ICTV.

## 1. Introduction

Viruses belonging to the *Orthobunyavirus* genus in the family *Peribunyaviridae* are arthropod-borne, lipid-enveloped viruses with three distinct genomic segments of single-stranded RNA (ssRNA) of negative polarity, denoted as large (L), medium (M), and small (S) [1]. The S segment encodes the nucleocapsid, the M segment encodes envelope glycoproteins, and the L segment encodes the polymerase protein [2]. Orthobunyaviruses take their name from the Bunyamwera virus (BUNV), which was originally isolated in 1943 from *Aedes* spp. mosquitoes in the Semliki Forest, Uganda [3]. The genus *Orthobunyavirus* is composed of a number of viruses, including some of medical and veterinary significance [4]. These viruses occur in many countries in Africa, the Americas, Europe, Asia, and Australia [4,5,6]. On the basis of the serological relationships of complement-fixing antibodies, as well as other assay results (hemagglutination inhibition, neutralizing antibody), the majority of orthobunyaviruses have been classified into 18 serogroups; some viruses remain ungrouped because they lack antigenic relatedness [4].

Among serogroups in the *Orthobunyavirus* genus, the Bunyamwera group is one of the most important because the serogroup members can cause febrile disease in both livestock and humans [5,7]. According to published data, the serogroup currently includes at least 26 different viruses: Birao virus (BIRV), Bozo virus (BOZOV), Bunyamwera virus (BUNV), Cache Valley virus (CVV), Cholul virus (CHLV), Fort Sherman virus (FSV), Kairi virus (KRIV), Lokern virus (LOKV), Main Drain virus (MDV), Northway virus (NORV), Potosi virus (POTV), Playas virus (PLAV), Santa Rosa virus (SARV), Shokwe virus (SHOV), Tensaw virus (TENV), Tlacotalpan virus (TLAV), Batai virus (BATV), Calovo virus (CVOV), Ngari virus (NRIV), Anadyr virus (ANADV), Germiston virus (GERV), Mboke virus (MBOV), Maguari virus (MAGV), Stanfield virus (STAV), Xingu virus (XINV), and Ilesha virus (ILEV) [7,8,9,10,11,12,13,14,15].

Traditionally, serological approaches have been used for the identification of orthobunyaviruses. However, a new classification of the order *Bunyavirales*, established in 2017 [16] and updated in 2019 by the International Committee on Taxonomy of Viruses (ICTV), defined species demarcation criteria within the *Orthobunyavirus* genus as a 96% or higher identity in the complete amino acid sequences of their L segments [17]. According to the current classification, the genus *Orthobunyavirus* includes 88 viral species, 12 of which contain members belonging to the Bunyamwera serogroup. These species are *Anadyr orthobunyavirus*, *Batai orthobunyavirus*, *Birao orthobunyavirus*, *Bunyamwera orthobunyavirus*, *Cache Valley orthobunyavirus*, *Fort Sherman orthobunyavirus*, *Ilesha orthobunyavirus*, *Kairi orthobunyavirus*, *Maguari orthobunyavirus*, *Main Drain orthobunyavirus*, *Potosi orthobunyavirus*, and *Tensaw orthobunyavirus*.

Regarding genovariants, the situation is as follows: *Batai orthobunyavirus* includes two genovariants (BATV and CVOV); *Bunyamwera orthobunyavirus* includes ten genovariants (BUNV, GERV, LOKV, MBOV, NRIV, NORV, SARV, SHOV, STAV, and XINV); *Cache Valley orthobunyavirus* includes three genovariants (CVV, CHLV, and TLAV); and *Maguari orthobunyavirus* includes two genovariants (MAGV and PLAV). In addition, some bunyaviruses are natural, intraspecies reassortants of different genovariants with proper names. Specifically, NRIV is a reassortant of BUNV and BATV within the *Bunyamwera orthobunyavirus* species [18,19]. CHLV is a reassortant of CVV and POTV within the *Cache Valley orthobunyavirus* species [14,19].

Such complicated relationships within the *Orthobunyavirus* genus, as well as the simultaneous use of serology-based classification, lead to difficulties in studying newly discovered orthobunyavirus genetic sequences. In order to eliminate emerging contradictions, we conducted a review of sequences of orthobunyaviruses belonging to the Bunyamwera serogroup (available in NCBI GenBank) and determined their phylogenetic relationships in accordance with the current ICTV report nomenclature.

## 2. Materials and Methods

Since the phenomenon of reassortment is quite common in the Bunyamwera serogroup, we considered only the strains/isolates for which complete sequences of all segments (S, M, and L) are known (Table S1). The alignment of nucleotide and amino acid sequences was performed using MEGA v.11 software (https://www.megasoftware.net/show_eua, free access, accessed on 12 April 2022) [20] using the MUSCLE algorithm [21]. Phylogenetic trees were reconstructed using maximum-likelihood estimation based on the general time-reversible (GTR) parametric model allowing gamma-distributed frequency variation between sites and a proportion of invariant sites in the sequence [22]. The GTR substitution model evaluated 24 models with various combinations of parameters of nucleotide substitution on the basis of maximum-likelihood fits and selected the best model among them. The model with the lowest BIC score (Bayesian information criterion) was considered to best describe the substitution pattern. A test for probable recombination was performed using the Recombination Detection Program (RDP) 4 beta 80 using eight methods provided by the software with the default settings (http://web.cbio.uct.ac.za/~darren/rdp.html, free access, accessed on 12 April 2022) [23].

## 3. Results

**Figure 1:**
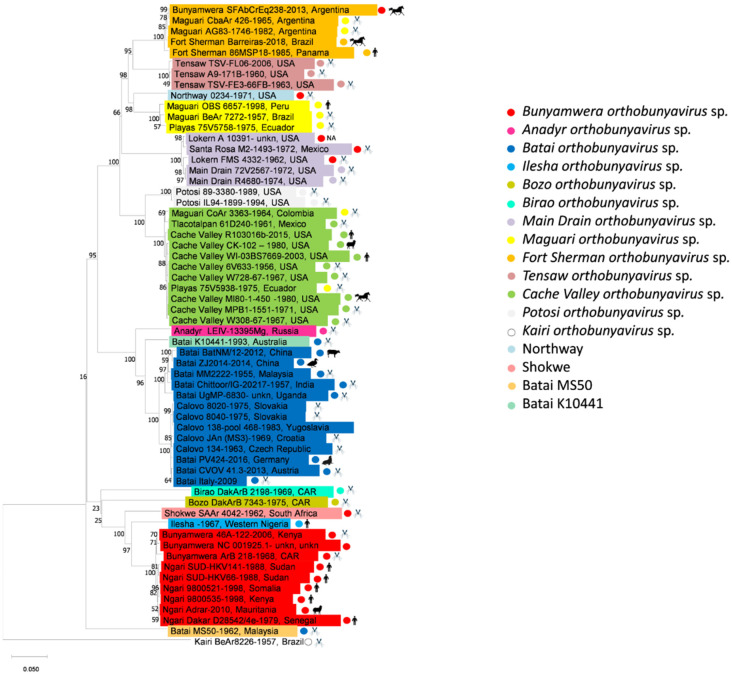
Phylogenetic tree of Bunyamwera serological group members based on L segment sequence (amino acid level). The tree was rooted to the genome of the La Crosse virus (EF485032). Alignments of amino acid sequences were performed using MEGA v.11 software using the MUSCLE algorithm. Phylogenetic trees were reconstructed using maximum-likelihood estimation based on the general time-reversible (GTR) parametric model allowing gamma-distributed frequency variation between sites and a proportion of invariant sites in the sequence. The robustness of the tree was tested using 1000 bootstrap replicates. The GTR substitution model evaluated 24 models with various combinations of parameters of nucleotide substitution on the basis of maximum-likelihood fits and selected the best model among them. Attribution to the orthobunyavirus species according to ICTV is indicated by a colored dot. The real attribution to the orthobunyavirus species is indicated by colored highlighting. Isolate source listed as an animal picture.

The phylogenetic analysis of the amino acid products of the L segments of full-length genomes showed the presence of 17 distinct clades, which can be considered to be different viral species within the Bunyamwera serogroup (Figure 1). Pairwise comparison of L segment full-length amino acid sequences enabled the attribution of viruses belonging to the Bunyamwera group to seventeen distinct viral species, in accordance with current ICTV demarcation criteria (Table S2). There is no doubt that ANADV, BIRV, BOZOV, ILEV, KRIV, POTV, and TENV should be considered different viral species. KRIV is the least related among known Bunyamwera serogroup members, with an aa identity ranging from 65.6% to 67.1%. Nevertheless, KRIV belongs to the serogroup according to its serological cross-reactions [8]. The full-genome sequences of three strains of KRIV are currently known: strain BeAr8226 (ac. no. NC_038738-NC_038740), strain TRVL 8900 (ac. no. MH484300-MH484302), and strain TR 8900 (ac. no. MH166874-MH166876); these strains were isolated in the 1950s. The TR 8900 sequence is actually a re-sequence of the TRVL 8900 strain that was completed in 2018.

Anadyr orthobunyavirus is represented in GenBank by sixteen full-genome sequences. All of them feature high levels of L segment aa identity (not less than 99.6%). *Birao orthobunyavirus* and *Bozo orthobunyavirus* are represented in GenBank by full-genome sequences of single isolates: BIRV DakArB 2198 (ac. no. NC_043650-NC_043652) and BOZOV DakArB 7343 (ac. no. NC_043653-NC_043655). These were isolated in 1969 and 1975, respectively.

*Ilesha orthobunyavirus* is represented in GenBank by three full-genome sequences of isolates: ILEV 8e (KC608149-KC608151), collected in the Central African Republic in 1964 [12]; ILEV KO/2 (MT272830-MT272832), isolated in Nigeria in 1967; and ILEV R5964, isolated in Nigeria in 1957 (NC_043585-NC_043587) [12].

*Potosi orthobunyavirus* is represented by two full-genome sequences of isolates: POTV 89-3380 (ac. no. MH484321-MH484323) and POTV IL94-1899 (ac. no. MF066368-MF066370). These were obtained in the USA in 1989 and 1994, respectively [24]. Tensaw orthobunyavirus is represented by three full-genome sequences of isolates TENV A9-171B (ac. no. MH484333-MH484335), TENV TSV-FE3-66FB (ac. no. NC_043546-NC_043548), and TENV TSV-FL06 (ac. nos. FJ943506, FJ943507, and FJ943509). These were isolated in the USA in 1960, 1963, and 2006 respectively [25]. The phylogenetic relationships of other species belonging to the Bunyamwera serogroup are more complicated (Figure 1).

### 3.1. Bunyamwera orthobunyavirus

Among the full-genome sequences designated as *Bunyamwera orthobunyavirus* in GenBank, only two strains/isolates should be attributed to *Bunyamwera orthobunyavirus* sp. according to current demarcation criteria: BUNV prototype strain from Uganda (ac. no. NC_001925-NC_001927), isolated in 1943 [26], and BUNV strain 46A-122-2006 (ac. no. MH484288-MH484290) from Kenya, isolated in 2006. In addition, six strains of NRIV could be attributed to *Bunyamwera orthobunyavirus* sp. as natural reassortants of BUNV and BATV, with 96% or more full L segment aa identity between BUNV and NRIV. These were NRIV strain 9800521 (ac. no. JX857325-JX857327) and NRIV strain 9800535 (ac. no. JX857328-JX857330), isolated in Somalia and Kenya in 1998; NRIV strain DakArD28542 (ac. no. JX857316-JX857318), isolated in 1979; NRIV strain SUD-HKV141 (ac. no. JX857322-JX857324) and NRIV strain SUD-HKV66 (ac. no. JX857319-JX857321), isolated in Sudan in 1988 [27]; and NRIV strain Adrar (ac. no. KJ716848-KJ716850), isolated in Mauritania in 2010 [28].

Other viruses defined as *Bunyamwera orthobunyavirus* sp. in the current ICTV report are not in good agreement with the current demarcation criteria, particularly strains SFCrEq231 (ac. no. KP063892-KP063894), SFBzEq232 (ac. no. KP063895-KP063897), and SFAbCrEq238 (ac. no. KP063898-KP063900). These were isolated in Argentina in 2013 and identified as BUNV strains [29] but should not be included as *Bunyamwera orthobunyavirus* sp. because they have less than 96% aa identity with the full-length L segment of the BUNV reference strain (ac. no. NC_001925).

The orthobunyaviruses NORV, LOKV, SARV, and SHOV also have less than 96% aa identity with the full-length L segment of the BUNV reference strain (ac. no. NC_001925). Therefore, they should not be attributed as genetic variants of *Bunyamwera orthobunyavirus* sp. NORV and SHOV are currently represented in GenBank by one full-genome sequence each: NORV strain 0234 (ac. no. MH484312-MH484314), collected in Alaska, U.S., in 1971 [30], and SHOV strain SAAr 4042 (ac. no. MH484330-MH484332), isolated in South Africa in 1962 [31]. According to the current demarcation criteria, both viruses could be considered independent species and may be designated as *Northway orthobunyavirus* sp. and *Shokwe orthobunyavirus* sp., respectively.

LOKV has two known isolates: LOKV isolate FMS4332 (ac. no. MG828823, MG820264, MG820265), isolated in the U.S. in 1962 [32], and LOKV isolate A10391 (ac. no. MH484303- MH484305) also isolated in the U.S. As mentioned above, LOKV could not be identified as a BUNV because it had less than 96% aa identity with *Bunyamwera orthobunyavirus* sp. However, LOKV shares more than 96% aa identity with SARV, which is represented in GenBank by one SARV isolate, M2-1493 (ac. no. MH484324-MH484326), isolated in Mexico in 1972 and unjustifiably classified as a genovariant of *Bunyamwera orthobunyavirus* sp. Thus, it would be even more logical to consider LOKV and SARV to be representatives of the same viral species, as discussed earlier [8].

Moreover, in accordance with the 96% aa identity criterion, LOKV and SARV should be considered *Main Drain orthobunyavirus* sp. genovariants. There are two known isolates of MDV with full-genome sequences: MDV strain R4680 (ac. no. MH484309-MH484311), isolated in the U.S. in 1974, and MDV strain 72V2567 (ac. no. MH484306-MH484308), isolated in the U.S. in 1972. The orthobunyaviruses LOKV, SARV, and MDV share at least 98.7% aa identity in the full L segment sequence. They should be considered genovariants of *Main Drain orthobunyavirus* sp. according to the current ICTV report. The L genes of the orthobunyaviruses GERV, MBOV, STAV, and XINV have not been sequenced completely. Thus, their taxonomic definitions could not be established correctly and require further study.

### 3.2. Batai orthobunyavirus

Currently, ten strains/isolates of BATV with complete genomes are available in GenBank. However, only eight of these sequences can be attributed to *Batai orthobunyavirus* sp. according to the current demarcation criteria. These strains/isolates are BATV strain NM/12 (ac. no. KJ187038-KJ187040), isolated in China in 2012 from cattle [33]; BATV strain Chittoor IG-20217 (ac. no. JX846598-JX846600), isolated in India in 1957 from *Anopheles barbirostris* sp.; BATV strain MM2222 (ac. no. JX846595-JX846597), isolated in Malaysia in 1955 from *Culex gelidus* sp.; BATV strain UgMP-6830 (ac. no. JX846601-JX846603), isolated in Uganda from *Aedes abnormalis* sp. [34]; BATV strain CVOV 41.3 (ac no KM507321-KM507323), isolated in Australia in 2013 from *Anopheles maculipennis* sp.; BATV strain Italy-2009 (ac. no. KC168046-KC168048), isolated in Italy in 2009 from *Anopheles maculipennis* complex mosquitoes [35]; BATV isolate PV424 (ac. no. MH299972-MH299974), isolated in Germany in 2016 from the seal species *Phoca vitulina* [36]; and BATV isolate ZJ2014 (ac. no. KU746869-KU746871), isolated in China in 2014 from the duck species *Cairina* *moschate* [37].

In addition to these, five strains/isolates of CVOV isolated from *Anopheles maculipennis* s.l. mosquitoes should be considered representatives of *Batai orthobunyavirus* sp.: CVOV strain 8020 (ac. no. KJ542630-KJ542632) and CVOV strain 840 (ac. no. KJ542633-KJ542635), both isolated in Slovakia in 1975; CVOV strain JAn (MS3) (ac. no. KJ542627- KJ542629), isolated in Croatia in 1969; CVOV strain 134 (ac. no. KJ542624-KJ542626), isolated in the Czech Republic in 1963 [9]; and CVOV strain 138-pool 468 (ac. no. KC608155-KC608157), isolated in Yugoslavia in 1983 in the territory of present-day Croatia [13]. BATV strains MS50 and K10441 should not be attributed to *Batai orthobunyavirus* sp. because they share less than 81% and 93% L segment aa identities with other strains/isolates of the species, respectively.

### 3.3. Cache Valley orthobunyavirus

As mentioned above, *Cache Valley orthobunyavirus* sp. Includes the genovariants CVV, CHLV, and TLAV. Eight strains of CVV, isolated mainly in the U.S. but also in Mexico in different years, are available in GenBank NCBI: CVV strain R103016b (ac. no. MK861965-MK861967), isolated in 2015 from humans [38]; CVV strain CK-102 (ac. no. KX100145-KX100147), isolated in 1980 from sheep; CVV strain WI-03BS7669 (ac. no. KX100151-KX100153), isolated in 2003 from humans; CVV strain 6V633 (ac. no. NC_043618-NC_043620), isolated in 1956 from *Culiseta inornate* mosquitoes; CVV strain W728-67 (ac. no. KX100136-KX100138), isolated in 1967 from *Aedes communis* mosquitoes; CVV strain MI80-1-450 (ac. no. KX100148-KX100150), isolated in 1980 from horses; CVV strain MPB1-1551 (ac. no. KX100142-KX100144), isolated in 1971 from *Psorophora confinnis* mosquitoes; and CVV strain W308-67 (ac. no. KX100139-KX100141), isolated in 1967 from *Aedes trivittatus* mosquitoes [35].

The only fully sequenced strain of Tlacotalpan virus available in GenBank is TLAV strain 61D240 (ac. no. KX100118-KX100120), isolated in Mexico in 1961 from *Culex titillans* mosquitoes [38]. To date, full-length CHLV L segments have not been obtained. Thus, it is not possible to attribute CHLV using current demarcation criteria. All the known CVV and TLAV strains share more than 96% L segment aa identity r and can be attributed to *Cache Valley orthobunyavirus* sp.

Moreover, MAGV strain CoAr 3363 (ac. no. KX100106-KX100108, isolated in Colombia in 1964 from *Aedes scapularis* mosquitoes) and PLAV strain 75V5938 (ac. no. KX100124-KX100126, isolated in Ecuador in 1975 from *Aedeomyia (Ochlerotatus) taeniorhynchus* mosquitoes) [39] should be attributed to *Cache Valley orthobunyavirus* sp. because they share at least 98.7% L segment aa identity with strains of that species.

### 3.4. Maguari orthobunyavirus

According to the ICTV report, *Maguari orthobunyavirus* sp. includes two genovariants: MAGV proper and PLAV. These are represented in GenBank by five and two complete genomes, respectively. However, only three of them could be defined as *Maguari orthobunyavirus* sp.: MAGV strain OBS 6657 (ac. no. KX100115-KX100117), isolated in Peru in 1998 from humans; MAGV strain BeAr 7272 (ac. no. KX100103-KX100105), isolated in Brazil in 1957 from mosquitoes; and PLAV strain 75V5758 (ac. no. KX100127-KX100129), isolated in Ecuador in 1975 from *Aedeomyia (Ochlerotatus) taeniorhynchus* mosquitoes [39].

Several strains should not be attributed to *Maguari orthobunyavirus* sp. because they share less than 96% aa identity with the L segment of the reference MAGV strain (BeAr 7272). These are MAGV strain CoAr 3363 (ac. no. KX100106-KX100108); MAGV virus strain CbaAr 426 (ac. no. KX100109-KX10011), isolated Argentina in 1965 from *Aedes albifasciatus* mosquitoes; MAGV strain AG83-1746 (ac. no. KX100112- KX100114), isolated in Argentina in 1982 from *Psorophora varinervis* mosquitoes; and PLAV strain 75V5938.

### 3.5. Fort Sherman orthobunyavirus

**Figure 2:**
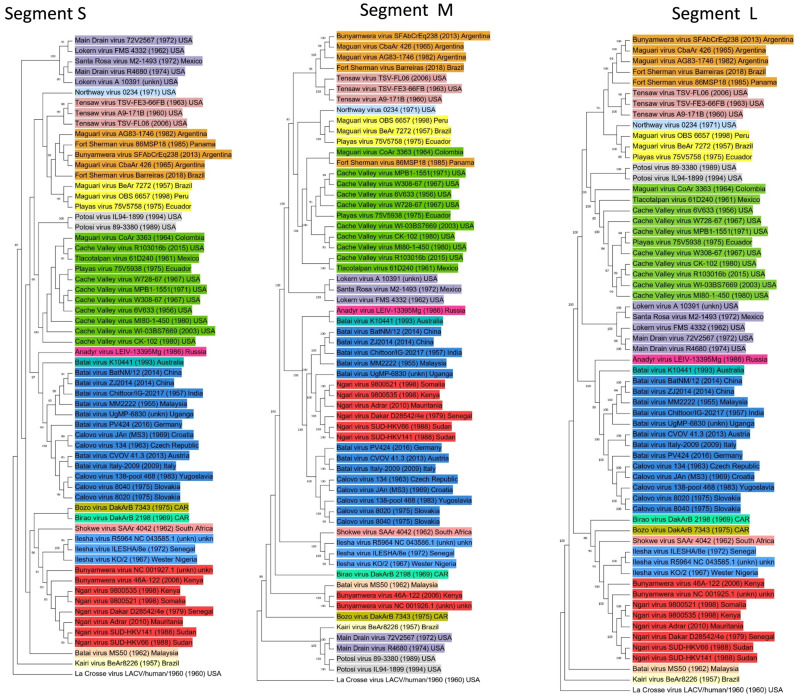
Phylogenetic trees for complete L, M, and S sequences of Bunyamwera serological group members (at the nucleotide level). The attribution to the orthobunyavirus species is indicated by colored highlighting as in Figure 1.

Two genovariants of *Fort Sherman orthobunyavirus* sp., FSV and Laguna Larga virus, are currently distinguished according to the ICTV. However, certain GenBank submissions for Laguna Larga virus sequences (ac. no. KX100109-KX10011) specified in the ICTV report actually belong to MAGV strain CbaAr 426 [16]. Besides the five strains/isolates of bunyaviruses available in GenBank, there are others that should be attributed to *Fort Sherman orthobunyavirus* sp.: FSV strain Barreiras (ac. no. MN379833- MN379835), isolated in Brazil in 2018 from horses [40]; FSV strain 86MSP18 (ac. no. NC_043615-NC_043617), isolated in Panama in 1985 from humans; MAGV strain CbaAr 426 (mentioned above); MAGV strain AG83-1746 (mentioned above); BUNV isolate SFCrEq231; BUNV isolate SFBzEq232; and BUNV isolate SFAbCrEq238 [29]. These share more than 96% aa identity of the full-length L segment. Dendrogram analyses of the L segment sequences at the aa and nucleic levels showed almost complete identity (Figure 1 and Figure 2). Minor differences were observed only in the position of viruses related to MDV, which may be an error in phylogenetic reconstruction.

The intra-species clusterization of Bunyamwera serogroup members into clades remains almost unchanged for all three segments. However, topological changes for some representatives were detected; this indicates the presence of interspecific reassortment within the Bunyamwera serological group. Apart from the two previously mentioned natural reassortants (NRIV, CHLV), we also identified three additional reassortment events. In segment M, FSV strain 86MSP18 forms a reliably supported clade related to CVV. In addition, MDV strain 72V2567 and MDV strain R4680 form a reliably supported clade related to two POTV strains (Figure 2). The reliability of these reassortment events was indirectly confirmed by the fact that the reassortant strains were isolated with some geographic proximity, either in the same state (in the U.S.) or in neighboring countries (Colombia, Venezuela). An analysis provided by RDP 4 beta 80 software showed an absence of probable recombination events in the L, M, and S segments.

The trees were rooted to the genome of the La Crosse virus (EF485030-EF485032). The alignment of nucleotide sequences was performed with MEGA v.11 software using the MUSCLE algorithm. Phylogenetic trees were reconstructed using maximum-likelihood estimation based on the general time-reversible (GTR) parametric model allowing gamma-distributed frequency variation between sites and a proportion of invariant sites in the sequence. The robustness of the tree was tested using 1000 bootstrap replicates. The GTR substitution model evaluated 24 models with various combinations of parameters of nucleotide substitution on the basis of maximum-likelihood fits and selected the best model among them. The accession numbers for the nucleotide sequences of the L, M, and S segments of the Bunyamwera serological group members are provided in Table S1. Attribution to the orthobunyavirus species according to the ICTV is indicated by a colored dot. The real attribution to the orthobunyavirus species is indicated by colored highlighting.

## 4. Discussion

The BUNV strains SFCrEq231 (ac. no. KP063892-KP063894), SFBzEq232 (ac. no. KP063895-KP063897), and SFAbCrEq238 (ac. no. KP063898-KP063900), as well as MAGV strain CbaAr 426 (ac. no. KX100109-KX10011) and MAGV strain AG83-1746 (ac. no. KX100112- KX100114), are *Fort Sherman orthobunyavirus* sp. In addition, NORV strain 0234 (ac. no. MH484312-MH484314) and SHOV strain SAAr 4042 (ac. no. MH484330-MH484332) were mistakenly defined as *Bunyamwera orthobunyavirus* sp. and should be considered separate species.

Both LOKV isolate FMS4332 (ac. nos. MG828823, MG820264, and MG820265) and LOKV isolate A10391 (ac. no. MH484303-MH484305), as well as SARV isolate M2-1493 (ac. no. MH484324-MH484326), should be considered *Main Drain orthobunyavirus* sp. It is noteworthy that LOKV and SARV share a bit more than 50% aa identity in the M segment with MDV strains while sharing 98.7% aa identity in the L segment and 99.6% aa identity in the S segment (with MDV), i.e., it is a reassortant. Therefore, it could be concluded that there is an undiscovered virus capable of forming reassortants with one of these three viruses that provided its M segment to LOKV and SARV or to MDV.

MAGV strain CoAr 3363 (ac. no. KX100106-KX100108) and PLAV strain 75V5758 (ac. no. KX100127-KX100129) should be considered *Cache Valley orthobunyavirus* sp. MAGV strain CbaAr 426 (ac. no. KX100109-KX10011) and MAGV strain AG83-1746 (ac. no. KX100112- KX100114) should be considered *Fort Sherman orthobunyavirus* sp. The latter two strains share 100% aa identity of their L segment proteins. However, there are minor differences in the aa sequences of other proteins.

BATV strain M150 [34] is considered a reference strain of the species. However, in accordance with current ICTV criteria, it belongs to a different viral species. Since from a historical point of view, the M50 strain was not the first BATV strain to be detected, it would be logical to leave the *Batai orthobunyavirus* sp. name for the main group and give a new species name to the M50 strain. This assumption is supported by Groseth et al., who determined that strain MS50 is, in fact, unrelated to BATV and likely represents a novel genotype in the genus *Orthobunyavirus* [34].

As mentioned above, BATV strain K10441 [6] also could not be attributed to *Batai orthobunyavirus* sp. This strain was isolated in Western Australia near Willare village. Perhaps it should be designated as a *Willare orthobunyavirus* sp.

Thus, there are sixty-four complete genomic sequences of Bunyamwera serogroup members currently represented in NCBI GenBank. In accordance with current ICTV demarcation criteria, these sequences should be divided into seventeen different viral species. In addition, species identifications were determined incorrectly for some of the viruses.

Specifically, of the sixteen sequences designated as *Bunyamwera orthobunyavirus* sp., three strains should be attributed to *Fort Sherman orthobunyavirus* sp.: SFCrEq231 (ac. no. KP063892-KP063894), SFBzEq232 (ac. no. KP063895-KP063897), and SFAbCrEq238 (ac. no. KP063898-KP063900). Two of the sixteen are different viral species: NORV (ac. no. MH484312-MH484314) and SHOV (ac. no. MH484330-MH484332). In addition, three of them should be attributed as *Main Drain orthobunyavirus* sp.: LOKV isolate A10391 (ac. no. MH484303-MH484305), LOKV isolate FMS 4332 (ac. nos. MG820264, MG828823, and MG696865), and SARV isolate M2-1493 (ac. no. MH484324-MH484326).

Among fifteen sequences classified as *Batai orthobunyavirus* sp., two should be attributed to different viral species: BATV isolate K10441 (ac. nos. KU661980, KU661984, and KU661991) and BATV strain MS50 (ac. no. NC_043579-NC_043581).

Among sequences classified as *Maguari orthobunyavirus* sp., three require reclassification: PLAV strain 75V5938 (ac. no. KX100124-KX100126), MAGV strain CoAr 3363 (ac. no. KX100109-KX100111), and MAGV strain AG83-1746 (ac. no. KX100112-KX100114). The first two should be attributed to *Cache Valley orthobunyavirus* sp. The third should belong to *Fort Sherman orthobunyavirus* sp.

We also defined M segment reassortment events between FSV strain 86MSP18 (ac. no. MH484294-MH484296) and CVV. Reassortments in M were also identified between MDV and POTV: MDV strain 72V2567 (ac. no. MH484306-MH484308) or MDV strain R4680 (ac. no. MH484309-MH484311) with POTV strain 89-3380 (ac. no. MH484321-MH484323) or POTV strain IL94-1899 (ac. no. NC_043645-NC_043647).

Therefore, interspecies reassortment events are possible between members of the Bunyamwera serological group [19]. Moreover, we obtained signs of reassortment of LOKV and SARV or MDV with an unknown virus. Thus, it can be assumed that orthobunyaviruses belonging to the Bunyamwera serological group are closely related viral species and are characterized by rather complicated phylogenetic relationships that need further clarification.

## Data Availability

Not applicable.

## Supplementary Materials

The following supporting information can be downloaded at https://www.mdpi.com/article/10.3390/v14061135/s1, Table S1: List of Bunyamwera serogroup sequences used in the study. Table S2: Similarity plot of L segment full-length amino acid sequences belonging to the Bunyamwera serogroup.
