# Supplementary material for: Current View on Genetic Relationships within the Bunyamwera Serological Group
Source: Viruses. 2022 May 25;14(6):1135. doi: 10.3390/v14061135 (PMC9227251; doi:10.3390/v14061135)
Supplement: Supplementary file 1 [file viruses-14-01135-s001.zip › Table_S1.pdf]

Table S1. List of Bunyamwera serogroup sequences used in the study.

№	Virus species (*ICTV*)	Virus species (in fact)	Virus name	Strain/isolate	Isolation region	Isolation source put genus and species names in italics	Isolation date	References
1	*Bunyamwera orthobunyavirus*	*Fort Sherman orthobunyavirus* (FSV)	Bunyamwera virus (BUNV)	SFAbCrEq238	Argentina	Horse	2013	KP063898-KP063900
2	*Bunyamwera orthobunyavirus*	*Fort Sherman orthobunyavirus* (FSV)	Bunyamwera virus (BUNV)	SFAbCrEq232	Argentina	Horse	2013	KP063895-KP063897
3	*Bunyamwera orthobunyavirus*	*Fort Sherman orthobunyavirus* (FSV)	Bunyamwera virus (BUNV)	SFCrEq231	Argentina	Horse	2013	KP063892-KP063894
4	*Bunyamwera orthobunyavirus*	*Bunyamwera orthobunyavirus*	Bunyamwera virus (BUNV)	46A-122	Kenya	*Aedes ochraceus*	2006	MH484288-MH484290
5	*Bunyamwera orthobunyavirus*	*Bunyamwera orthobunyavirus*	Bunyamwera virus (BUNV)	unknown	unknown	unknown	unknown	NC001925-NC001927
6	*Bunyamwera orthobunyavirus*	*Bunyamwera orthobunyavirus*	Ngari virus (NRIV)	9800521	Somalia	Human	1998	JX857325-JX857327
7	*Bunyamwera orthobunyavirus*	*Bunyamwera orthobunyavirus*	Ngari virus (NRIV)	9800535	Kenya	Human	1998	JX857328-JX857330
8	*Bunyamwera orthobunyavirus*	*Bunyamwera orthobunyavirus*	Ngari virus (NRIV)	Adrar	Mauritania	Small ruminant	2010	KJ716848-KJ716850

9	*Bunyamwera orthobunyavirus*	*Bunyamwera orthobunyavirus*	Ngari virus (NRIV)	Dakar D28542/4e	Senegal	Human	1979	KC608152-KC608154
10	*Bunyamwera orthobunyavirus*	*Bunyamwera orthobunyavirus*	Ngari virus (NRIV)	SUD-HKV66	Sudan	Human	1988	JX857319-JX857321
11	*Bunyamwera orthobunyavirus*	*Bunyamwera orthobunyavirus*	Ngari virus (NRIV)	SUD-HKV141	Sudan	Human	1988	JX857322-JX857324
12	*Bunyamwera orthobunyavirus*	*Northway orthobunyavirus* *	Northway virus (NORV)	0234	USA	Aedes sp.	1971	MH484312-MH484314
13	*Bunyamwera orthobunyavirus*	Shokwe *orthobunyavirus* *	Shokwe virus (SHOV)	SAAr 4042	South Africa	Aedes cumminsii	1962	MH484330-MH484332
14	*Bunyamwera orthobunyavirus*	*Main Drain ortobunyavirus*	Lokern virus (LOKV)	A 10391	USA	unknown	unknown	MH484303-MH484305
15	*Bunyamwera orthobunyavirus*	*Main Drain ortobunyavirus*	Lokern virus (LOKV)	FMS 4332	USA	Culex tarsalis	1962	MG820264, MG828823, MG696865
16	*Bunyamwera orthobunyavirus*	*Main Drain ortobunyavirus*	Santa Rosa virus (SARV)	M2-1493	Mexico	Ochlerotatus angustivittatus	1972	MH484324-MH484326
17	*Main Drain ortobunyavirus*	*Main Drain ortobunyavirus*	Main Drain virus (MDV)	72V2567	USA	Aedes vexans	1972	MH484306-MH484308
18	*Main Drain ortobunyavirus*	*Main Drain ortobunyavirus*	Main Drain virus (MDV)	R4680	USA	Anopheles freeborni	1974	MH484309-MH484311
19	*Batai orthobunyavirus*	*Willare orthobunyavirus* *	Batai virus (BATV)	K10441	Australia	Culex annulirostris	1993	KU661980, KU661984, KU661991
20	*Batai orthobunyavirus*	*Batai orthobunyavirus*	Batai virus (BATV)	BatNM/12	China	Cattle	2014	KJ187038-KJ187040

21	*Batai orthobunyavirus*	*Batai orthobunyavirus*	Batai virus (BATV)	ZJ2014	China	Muscovy duck	2014	KU746869-KU746871
22	*Batai orthobunyavirus*	*Batai orthobunyavirus*	Batai virus (BATV)	MM2222	Malaysia	Culex gelidus	1955	JX846595-JX846597
23	*Batai orthobunyavirus*	*Batai orthobunyavirus*	Batai virus (BATV)	Chittoor/IG-20217	India	Anopheles barbirostris	1957	JX846598-JX846600
24	*Batai orthobunyavirus*	*Batai orthobunyavirus*	Batai virus (BATV)	UgMP-6830	Uganga	Aedes abnormalis	unknown	JX846601-JX846603
25	*Batai orthobunyavirus*	*Batai orthobunyavirus*	Batai virus (BATV)	CVOV 41.3	Austria	Anopheles maculipennis	2013	KM507321-KM507323
26	*Batai orthobunyavirus*	*Batai orthobunyavirus*	Batai virus (BATV)	Italy-2009	Italy	Mosquito	2009	KC168046-KC168048
27	*Batai orthobunyavirus*	*Batai orthobunyavirus*	Batai virus (BATV)	PV424	Germany	Phoca vitulina	2016	MH299972-MH299974
28	*Batai orthobunyavirus*	MS50 *orthobunyavirus*	Batai virus (BATV)	MS50	Malaysia	Aedes curtipes	1962	NC_043579-NC_043581
29	*Batai orthobunyavirus*	*Batai orthobunyavirus*	Calovo virus (CVOV)	134	Czech Republic	Anopheles maculipennis	1963	KJ542624-KJ542626
30	*Batai orthobunyavirus*	*Batai orthobunyavirus*	Calovo virus (CVOV)	JAn (MS3)	Croatia	Anopheles maculipennis	1969	KJ542627-KJ542629
31	*Batai orthobunyavirus*	*Batai orthobunyavirus*	Calovo virus (CVOV)	138-pool 468	Yugoslavia	unknown	1983	KC608155-KC608157
32	*Batai orthobunyavirus*	*Batai orthobunyavirus*	Calovo virus (CVOV)	8020	Slovakia	Anopheles maculipennis	1975	KJ542630-KJ542632

33	*Batai orthobunyavirus*	*Batai orthobunyavirus*	Calovo virus (CVOV)	8040	Slovakia	Anopheles maculipennis	1975	KJ542633-KJ542635
34	*Cache Valley orthobunyavirus*	*Cache Valley orthobunyavirus*	Cache Valley virus (CVV)	6V633	USA	Culiseta inornata	1956	NC_043618-NC_043620
35	*Cache Valley orthobunyavirus*	*Cache Valley orthobunyavirus*	Cache Valley virus (CVV)	W728-67	USA	Aedes communis	1967	KX100136-KX100138
36	*Cache Valley orthobunyavirus*	*Cache Valley orthobunyavirus*	Cache Valley virus (CVV)	MPB1-1551	USA	Psorophora confinnis	1971	KX100142-KX100144
37	*Cache Valley orthobunyavirus*	*Cache Valley orthobunyavirus*	Cache Valley virus (CVV)	W308-67	USA	Aedes trivittatus	1967	KX100139-KX100141
38	*Cache Valley orthobunyavirus*	*Cache Valley orthobunyavirus*	Cache Valley virus (CVV)	CK-102	USA	Sheep	1980	KX100145-KX100147
39	*Cache Valley orthobunyavirus*	*Cache Valley orthobunyavirus*	Cache Valley virus (CVV)	R103016b	USA	Human	2015	MK861965-MK861967
40	*Cache Valley orthobunyavirus*	*Cache Valley orthobunyavirus*	Cache Valley virus (CVV)	WI-03BS7669	USA	Human	2003	KX100151-KX100153
41	*Cache Valley orthobunyavirus*	*Cache Valley orthobunyavirus*	Cache Valley virus (CVV)	MI80-1-450	USA	Horse	1980	KX100148-KX100150
42	*Cache Valley orthobunyavirus*	*Cache Valley orthobunyavirus*	Tlacotalpan virus (TLAV)	61D240	Mexico	Mansonia titillans	1961	MH484342-MH484344
43	*Maguari orthobunyavirus*	*Cache Valley orthobunyavirus*	Playas virus (PLAV)	75V5938	Ecuador	Aedeomyia taeniorhynchus	1975	KX100124-KX100126
44	*Maguari orthobunyavirus*	*Maguari orthobunyavirus*	Playas virus (PLAV)	75V5758	Ecuador	Aedeomyia taeniorhynchus	1975	KX100127-KX100129

45	*Maguari orthobunyavirus*	*Cache Valley orthobunyavirus*	Maguari virus (MAGV)	CoAr 3363	Colombia	Aedes scapularis	1964	KX100106-KX100108
46	*Maguari orthobunyavirus*	*Maguari orthobunyavirus*	Maguari virus (MAGV)	OBS 6657	Peru	Human	1998	KX100115-KX100117
47	*Maguari orthobunyavirus*	*Maguari orthobunyavirus*	Maguari virus (MAGV)	BeAr 7272	Brazil	Mosquito	1957	KX100103-KX100105
48	*Maguari orthobunyavirus*	*Fort Sherman orthobunyavirus*	Maguari virus (MAGV)	AG83-1746	Argentina	Psorophora varinervis	1982	KX100112-KX100114
49	*Maguari orthobunyavirus*	*Fort Sherman orthobunyavirus*	Maguari virus (MAGV)	CbaAr 426	Argentina	Aedes albifasciatus	1965	KX100109-KX100111
50	*Fort Sherman orthobunyavirus*	*Fort Sherman orthobunyavirus*	Fort Sherman virus (FSV)	Barreiras	Brazil	Horse	2018	MN379833-MN379835
51	*Fort Sherman orthobunyavirus*	*Fort Sherman orthobunyavirus*	Fort Sherman virus (FSV)	86MSP18	Panama	Human	1985	MH484294-MH484296
52	*Tensaw orthobunyavirus*	*Tensaw orthobunyavirus*	Tensaw virus (TENV)	TSV-FL06	USA	Mosquito	2006	FJ943506, FJ943507, FJ943509
53	*Tensaw orthobunyavirus*	*Tensaw orthobunyavirus*	Tensaw virus (TENV)	TSV-FE3-66FB	USA	Mosquito	1963	NC_043546-NC_043548
54	*Tensaw orthobunyavirus*	*Tensaw orthobunyavirus*	Tensaw virus (TENV)	A9-171B	USA	Anopheles crucians	1960	MH484333-MH484335
55	*Potosi orthobunyavirus*	*Potosi orthobunyavirus*	Potosi virus (POTV)	89-3380	USA	Aedes albopictus	1989	MH484321-MH484323
56	*Potosi orthobunyavirus*	*Potosi orthobunyavirus*	Potosi virus (POTV)	IL94-1899	USA	Aedes albopictus	1994	NC_043645-NC_043647

57	*Anadyr orthobunyavirus*	*Anadyr orthobunyavirus*	Anadyr virus (ANADV)	LEIV-13395Mg	Russia	Aedes sp.	1986	NC_055199-NC_055201
58	*Birao orthobunyavirus*	*Birao orthobunyavirus*	Birao virus (BIRV)	DakArB 2198	CAR	Anopheles pharoensis	1969	MH484282-MH484284
59	*Bozo orthobunyavirus*	*Bozo orthobunyavirus*	Bozo virus (BOZOV)	DakArB 7343	CAR	Aedes opok	1975	NC_043653-NC_043655
60	*Ilesha orthobunyavirus*	*Ilesha orthobunyavirus*	Ilesha virus (ILEV)	ILESHA/8e	Senegal	Human	1972	KC608149-KC608151
61	*Ilesha orthobunyavirus*	*Ilesha orthobunyavirus*	Ilesha virus (ILEV)	R5964	Nigeria	Human	1957	NC_043585-NC_043587
62	*Ilesha orthobunyavirus*	*Ilesha orthobunyavirus*	Ilesha virus (ILEV)	KO/2	Western Nigeria	Human	1967	MT272830-MT272832
63	*Kairi orthobunyavirus*	*Kairi orthobunyavirus*	Kairi virus (KRIV)	BeAr8226	Brazil	Ochlerotatus scapularis	1957	NC_038738-NC_038740
64	*La Crosse orthobunyavirus*	*La Crosse orthobunyavirus*	La Crosse virus (LACV)	LACV/human/1960	USA	Human	1960	EF485030-EF485032
